# Contact Pressure Level Indication Using Stepped Output Tactile Sensors

**DOI:** 10.3390/s16040511

**Published:** 2016-04-09

**Authors:** Eunsuk Choi, Onejae Sul, Juyoung Kim, Kyumin Kim, Jong-Seok Kim, Dae-Yong Kwon, Byong-Deok Choi, Seung-Beck Lee

**Affiliations:** 1 Department of Electronic Engineering, Hanyang University, 222 Wangsimni-ro, Seongdong-gu, Seoul 133-791, Korea; silver77@hanyang.ac.kr (E.C.); jywhat@hanyang.ac.kr (J.K.); skylab7@hanyang.ac.kr (K.K.); kjskim383@hanyang.ac.kr (J.-S.K.); dy.kwon87@gmail.com (D.-Y.K.); bdchoi@hanyang.ac.kr (B.-D.C.); 2 Institute of Nano Science and Technology, Hanyang University, 222 Wangsimni-ro, Seongdong-gu, Seoul 133-791, Korea; ojsul@hanyang.ac.kr

**Keywords:** tactile sensor, tactile sensor array, spatially digitized electrode, stepped output characteristics

## Abstract

In this article, we report on a novel diaphragm-type tactile pressure sensor that produces stepwise output currents depending on varying low contact pressures. When contact pressures are applied to the stepped output tactile sensor (SOTS), the sensor’s suspended diaphragm makes contact with the substrate, which completes a circuit by connecting resistive current paths. Then the contact area, and therefore the number of current paths, would determine the stepped output current produced. This mechanism allows SOTS to have high signal-to-noise ratio (>20 dB) in the 3–500 Hz frequency range at contact pressures below 15 kPa. Moreover, since the sensor’s operation does not depend on a material’s pressure-dependent electrical properties, the SOTS is able to demonstrate high reproducibility and reliability. By forming a 4 × 4 array of SOTS with a surface bump structure, we demonstrated shear sensing as well as surface (1 × 1 cm^2^) pressure mapping capabilities.

## 1. Introduction

Recently, tactile pressure sensors fabricated over an arbitrary substrate, or artificial skins, are being developed for possible applications ranging from touch sensitive robotic interfaces to biomedical instrumentation [1,2,3,4]. As pressure sensors, they are required to have high pressure sensitivity, low pressure threshold [5,6] and wide sensing range [7,8]. At the same time, the sensors are required to have uniformity and reliability with high spatial resolution [9], and they consist a system with multiple arrays of these sensors. In addition to these functional requirements, for biomimetic operation, they aim to mimic the function of the human skin, emulating the human mechanoreceptors having higher sensitivity for change in relative magnitude of pressure than absolute values for higher differential pressure sensitivity at very low pressure ranges [10]. In recent years, many pressure sensors have been reported that utilize various aspects of nanomaterials and microstructures to produce high pressure sensitivity showing output signals with amplitude proportional to the strength of the applied stimulus [11,12,13,14,15,16,17,18,19,20,21,22]. Although the reported sensors demonstrate high sensitivity, most lack actual applicability since sensors based on nanomaterials, such as carbon nanotubes or graphitic oxides, have issues with uniformity and reproducibility [23,24]. Especially, capacitance type sensors have issues with cross-talk and noise when measuring low pressure levels at high spatial resolution [25,26].

In this article, we demonstrate the operation of a stepped output tactile sensor (SOTS) which produces stepwise changes in current outputs depending on analog tactile pressure inputs. The sensor’s operation, in principle, does not depend on the pressure dependent resistivity of an active sensing material but mostly on the geometry and layout of the contact electrodes. The basic concept follows from a previous report where spatially separated contact electrodes were used to detect consecutive deflection of a diaphragm depending on the applied pressure. The output from a particular electrode corresponded to a specific applied pressure, producing a digitized output signal [27]. One drawback in this sensor was that the number of output electrodes had to be increased if the pressure detection range and resolution were to be increased, making the sensor impractical for array type sensor application which would require too many electrodes for integration. Here, we reduced the output electrode numbers to two per sensor while still maintaining the basic operational concept by utilizing a parallel distribution of resistive current paths. When a suspended diaphragm, with a conducting layer, deflects with tactile pressure, it makes contact with the substrate, and completes a circuit by connecting a number of resistive current paths. Therefore the resulting output current reflects the level of applied pressure. Although the sensing mechanism is simple, SOTS produces a highly reliable output current with the number and the spacing of the current paths determining the detection resolution and pressure range. The sensor demonstrates high reproducibility with high signal-to-noise ratio (SNR) of more than 20 dB in 3~500 Hz frequency range. Also by forming a 4 × 4 SOTS array, it was possible to demonstrate detection of spatially distributed contact pressures over a 1 cm^2^ surface area. With further developments it will be possible to utilize the SOTS system for robotic tactile interfaces and electric skin prosthesis.

## 2. Stepped Output Tactile Sensor

### 2.1. Concepts and Operating Mechanism

**Figure 1:**
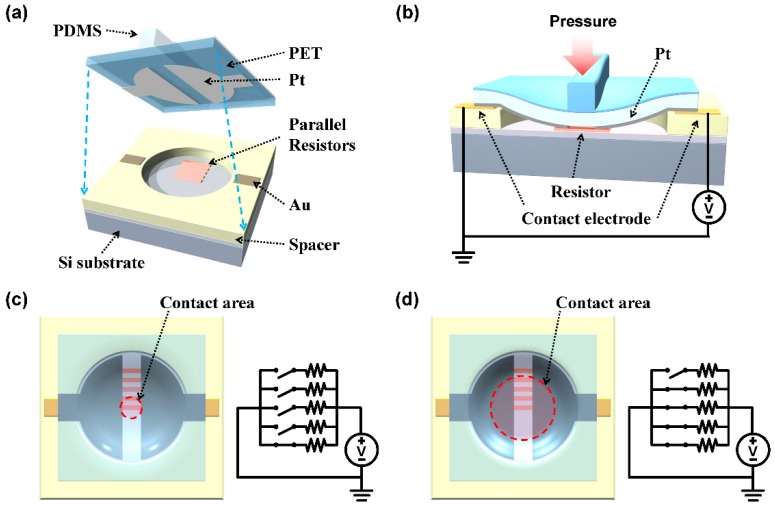
The schematic illustration showing the operating mechanism of the stepped output tactile sensor (SOTS): (**a**) The schematic diagram of SOTS; (**b**) the 3D cross-sectional image under applied pressure, showing short-circuited multiple resistors. The contact areas and equivalent circuits of SOTS when small (**c**) or large (**d**) pressure was applied.

Figure 1a shows a schematic diagram of the device. It consists mainly of two parts, the upper diaphragm layer and the bottom substrate. The upper layer consists of a polyethylene terephthalate (PET) film with a polydimethylsiloxane (PDMS) ridge structure on top [27,28,29]. On the bottom of the PET film, Pt contact electrodes were fabricated to act as the two contact electrodes. On the substrate, parallel resistors were placed from the center of the spacer defined pit outwards, which will act as digitized current paths.

When tactile pressure is applied, the upper PET diaphragm deflects and makes contact with the central resistor at a threshold pressure (Figure 1b), completing a circuit with a corresponding current output (Figure 1c). Initial tactile pressures below the threshold level will not be detected by the sensor, since the threshold pressure will be the minimum pressure level for the intended tactile sensing application. This will contribute to reducing sensor power consumption because no power will be consumed during tactile pressures below threshold. If required, the threshold pressure can be lowered by reducing the spacer thickness or by increasing the diaphragm diameter. Increasing the pressure further results in more resistor current paths being contacted in sequence, which leads to a stepwise increase in current (Figure 1d). Since each value of current plateau corresponds to a designated range of contact pressure, the distance between the resistors determines the pressure detection resolution of the sensor. The closer they are, the higher the resolution.

### 2.2. Fabrication Process

**Figure 2:**
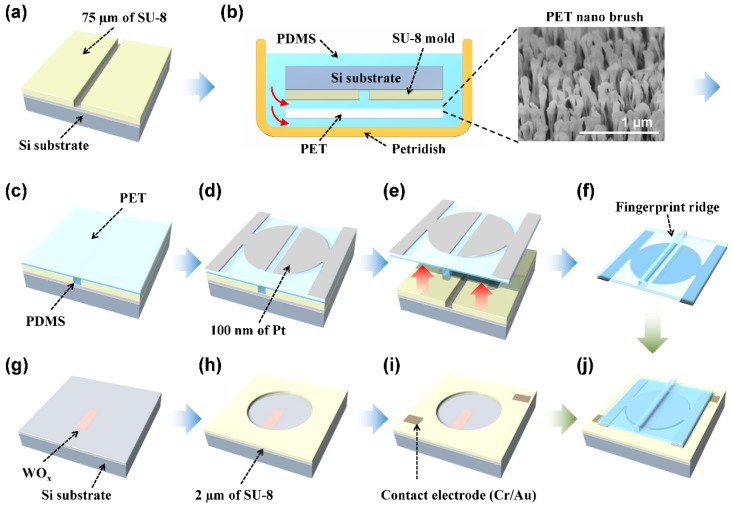
The fabrication process of SOTS: (**a**) SU-8 master for the top ridge structure; (**b**) PDMS molding using PET nanobrushs with inset showing the SEM image; (**c**) complete curing; (**d**) deposition of the top electrode; (**e**) peel-off; (**f**) fabricated top layer; (**g**) deposition of spatially digitized resistor; (**h**) SU-8 spacer formation; (**i**) deposition of contact electrodes; and (**j**) bonding the top layer and the bottom substrate.

Figure 2 shows illustrations of the device fabrication process. For the upper layer fabrication, a mold-transfer technique was used. A 75 μm-thick SU-8 master layer was patterned to form a negative of the top ridge structure (Figure 2a). Then the PET film, that will form the diaphragm, was placed on top of the SU-8 master and was laid in a Petri dish, after which PDMS was poured on top (Figure 2b). The SU-8 facing surface of the PET was treated with reactive ion etching (performed at 5 mTorr of pressure, 30 sccm of CF_4_ gas flow, 100 W of RF power, for 10 min) to form nanobrush structures. The PET nanobrushes, shown in the SEM image of Figure 2b, promoted the adhesion of the PDMS to PET by increasing the contact surface area between PET film and PDMS layer. The PDMS solution percolated into the 30 μm gap between the PET film and the master to form the top skin of the sensor (Figure 2c). After curing the PDMS, 100 nm thick Pt electrodes, with a gap of 50 μm, were formed using optical lithography and sputter deposition (Figure 2d). Finally, the top layer was peeled off (Figure 2e). The bottom substrate fabrication process begins with the defining of WO_x_ spatially digitized parallel resistors on the oxidized Si substrate (Figure 2g). The resistors were formed by defining the pattern using electron beam lithography in PMMA and sputtering W (50 nm thickness) in oxygen atmosphere. The deflection pit (2, 4, 8 mm diameters) was defined using optical lithography in 2-μm-thick SU-8 negative resist which was used as the spacer layer (Figure 2h). 

The electrodes (20 nm Cr/100 nm Au) used to make contact with the top Pt layer were patterned on the SU-8 surface by optical lithography and thermal evaporation (Figure 2i). Finally the fabricated top layer and bottom substrate were combined using conducting paste (Figure 2j).

**Figure 3:**
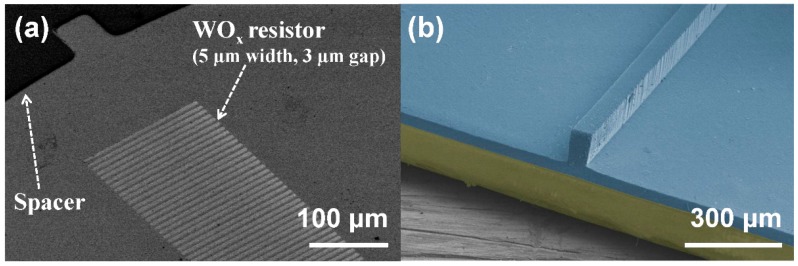
The SEM images of SOTS: (**a**) The SEM image of spatially digitized WO_x_ resistors and SU-8 spacer with 2 µm height and 2 mm diameter; (**b**) the SEM image of top structure composed of the PDMS ridge and the PET substrate, with false coloring.

Figure 3a shows a SEM image of the spacer and the WO_x_ parallel resistors. The resistors were 150 μm in length, with 5 μm width. Multiple resistors were arranged with 3 μm gaps starting from pit center to edge. Figure 3b shows a SEM image of the top layer with a 75 μm high and 50 μm wide ridge structure. The resistivity of WO_x_ resistors were 4.5 × 10^−4^ Ωm, which formed 90 kΩ resistors. The full size of the devices was 2 × 2 cm^2^ in area.

### 2.3. Pressure Detection Characteristics

**Figure 4:**
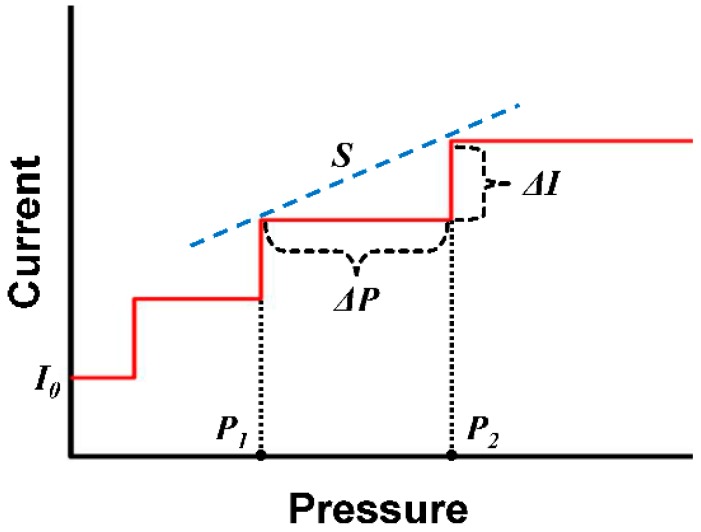
The graphical definition of sensitivity of the SOTS.

In general, the sensitivity of a pressure sensor is defined as *d*(*A_Output_*/*A*_0_)/*dP*, where *A_Output_* is the output quantity of a pressure sensor such as current, resistance or capacitance, and *A*_0_ is its initial value. However in our case, since the output levels were in steps, the definition was inadequate. Thus, we have modified the definition to fit our sensor operation as follows:

(1) $$S=\frac{\Delta I}{I_{0}}\times \frac{1}{\Delta P}=\frac{R_{0}}{R_{resistor}}\times \frac{1}{\Delta P}=\frac{1}{R_{resistor}\times \Delta P}\times R_{0}$$

where, *I*_0_ and *R*_0_ refer to the sensor’s off-state current and resistance, and *R_resistor_* refers to the resistance of the parallel resistors (see Figure 4). Δ*I* is the step of current variation due to individual resistors, which were 11 mA (=1 V/90 kΩ) when the 1 V bias voltage was applied. Δ*P* is the pressure increment required to activate the next resistor, which depends on the spacing between the resistors. From Equation (1), it is clear that higher sensitivity can be achieved by reducing either the resistance of a resistor or their spacing. If the resistance due to the resistors were minimized, then the total resistance of the sensor would be dominated by the resistance of Au and Pt electrodes, which would not be desirable. Thus reduction of the spacing, and therefore, the pressure variation was more desirable.

Δ*P* can be defined analytically starting from the diaphragm model [30]. We use the diaphragm model as a reference for the device concept and so the model does not correspond faithfully to the actual behavior of the diaphragm. But we may analyze the behavior of our device, such as sensitivity and its dependence on geometrical parameters, based on this model. The deflection of the diaphragm *w* at the distance *r* from the center of a diaphragm is given as:

(2) $$w=k\frac{P{\left(1-\nu \right)}^{2}}{Et^{3}}{\left({\left(\frac{d}{2}\right)}^{2}-r^{2}\right)}^{2}$$

where *E* is the Young’s modulus, *ν* is the Poisson’s ration, *t* is the thickness, and *d* is the diameter of the diaphragm, with a proportionality constant *k*. If the diaphragm touches the bottom of the pit, then the radius of contact *r_contact_* is determined by the pressure *P*:

(3) $$P=\frac{Et^{3}h}{k{\left(1-\nu \right)}^{2}}\times {\left({\left(\frac{d}{2}\right)}^{2}-r_{contact}{}^{2}\right)}^{-2}$$

here *h* is the height of the pit. Assume that *P_n_* is the pressure when the diaphragm makes contact with the *n*th resistor at *r_n_* and that *P_n+1_* for the next resistor at *r*_*n*+1_. Then the distance between the *n*th and the next resistor is Δ*r* = *r_n+_*_1_ – *r_n_*. Then the pressure variation is Δ*P* = *P_n+_*_1_ – *P_n_* = *P_n+_*_1_(1 – *P_n_*/*P_n+_*_1_) determined by the pressure ratio, *P_n_*/*P_n+_*_1_:

(4) $$\frac{P_{n}}{P_{n+1}}={\left(\frac{{\left(\frac{d}{2}\right)}^{2}-r_{n+1}{}^{2}}{{\left(\frac{d}{2}\right)}^{2}-r_{n}{}^{2}}\right)}^{2}={\left(1-\frac{{\left(r_{n}+\Delta r\right)}^{2}-r_{n}{}^{2}}{{\left(\frac{d}{2}\right)}^{2}-r_{n}{}^{2}}\right)}^{2}$$

From Equation (4), minimization of Δ*r* leads to reduction of Δ*P* and maximization of sensitivity *S*. Therefore, the pressure sensitivity of SOTS can be controlled by adjusting the spacing between neighboring resistors. This feature was well demonstrated by the following experimental results.

**Figure 5:**
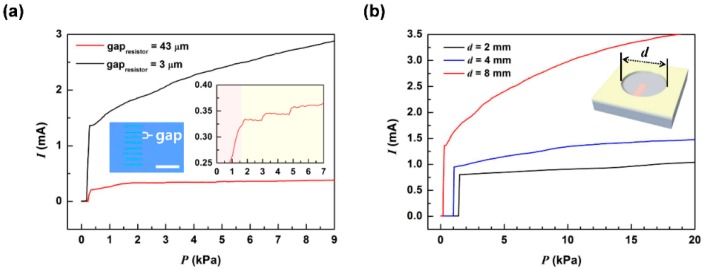
The basic operating characteristics of SOTS. (**a**) the pressure dependent output characteristics of SOTS1 (resistor spacing = 43 µm) and SOTS2 (resistor spacing = 3 µm) with identical pit diameter of 8 mm with the inset showing the optical image of SOTS1 and expanded output characteristics of SOTS1; and (**b**) the pressure dependent current measurements of SOTS2 depending on the 2, 4, 8 mm of pit diameter.

Figure 5a shows the contact pressure dependent output of SOTSs with different resistor spacing, 43 μm (SOTS1) and 3 μm (SOTS2), with identical pit diameter of 8 mm. Having the same diameter means that the diaphragm deflection in both sensors would be the same for equally applied contact pressures. It can be seen that the change in output current, with applied pressure, of SOTS2 was higher than SOTS1. The change in output current for the same change in applied pressure was ~6 times higher for SOTS2. This was due to the higher density of resistors (6 times more) making contact in SOTS2 than in SOTS1 for the same applied pressures. At the moment of contact, the output current from SOTS2 shows a jump from 0 to 1.35 mA at 1 V supply voltage, which means that the initial contact signal was made with 39 to 41 resistors, making the diameter of the contact area 312~332 μm. The initial rise in the output current of SOTS1, up to 1.6 kPa of applied pressure, was attributed to about 6 to 8 resistors being contacted at the same time with the contact area gradually increasing. Beyond this pressure, steps in output currents were observed with increase in pressure, as highlighted in the inset of Figure 5a. Clear steps of ~15 μA were observed at 3 kPa and 5 kPa indicating that the sensors may be designed to produce differential current steps at predesignated applied pressures. This stepped output was not observed for SOTS2 due to the higher number of resistors making contact which resulted in a continuous rise in the output with applied pressures, similar to conventional pressure sensors relying on piezoresistive outputs of their active sensing materials. Therefore, by varying the spacing between the resistors, SOTS was able to change its output mode from stepped to continuous.

The pressure sensitivity may also be controlled by the pit diameter. Figure 5b shows the pressure dependent output current of SOTS2 type devices with different pit diameters. Since the diameter of the pit determines the pressure dependent deflection depth of the PET diaphragm, it will determine the pressure sensitivity.

The threshold pressures and sensitivities were 0.2, 1.0, 1.4 kPa and 17.8, 5.7, 1.8 kPa^−1^ for SOTS2 device with 8, 4, 2 mm pit diameters around 5 kPa, respectively. The result implies that the sensor’s pressure sensing range can be tailored by changing the pit diameter. The sensitivity of SOTS2 device around 5 kPa compares with the high sensitivity values of previously reported sensors: 0.15 kPa^−1^ [12]; 0.2 kPa^−1^ [14]; 0.14 kPa^−1^ [17], 5.54 kPa^−1^ [22].

### 2.4. Repeatability Test & Detection of Vibration and Grating

**Figure 6:**
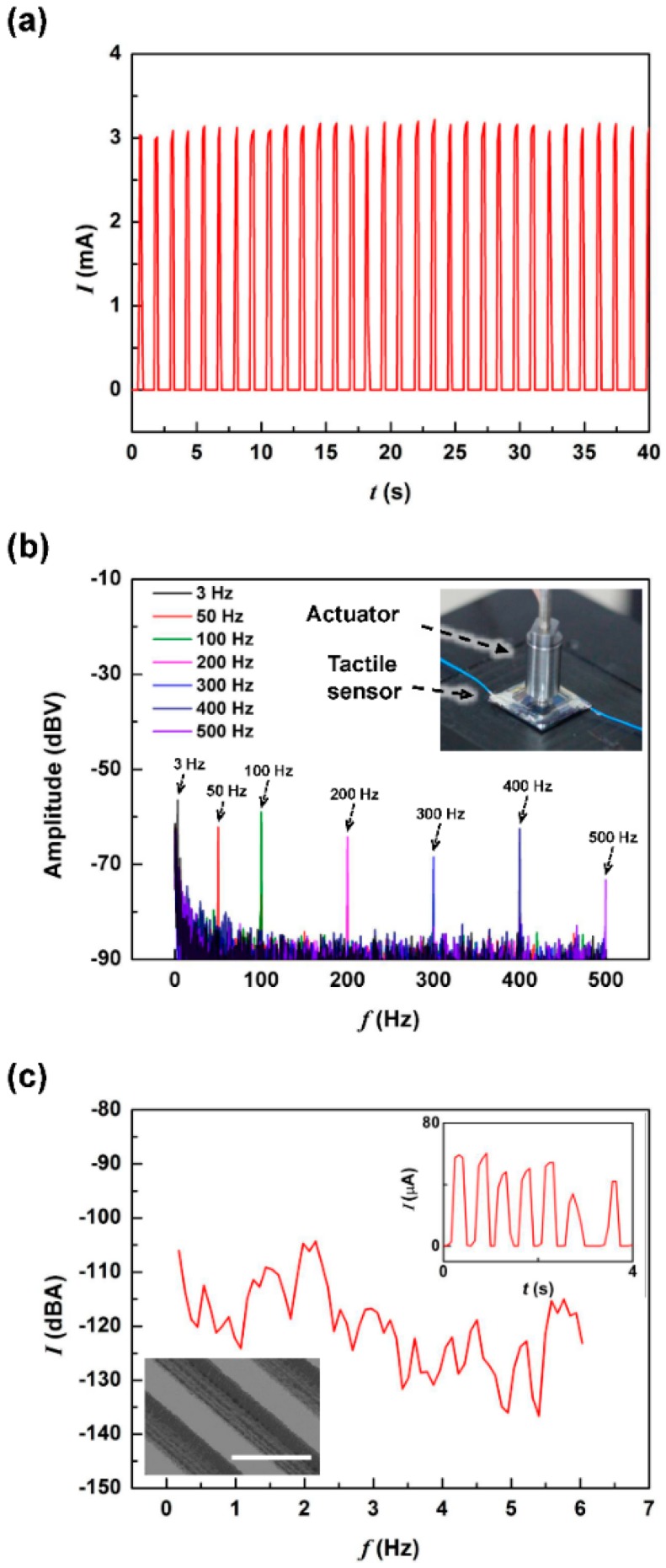
The repeatable pressure sensing operation SOTS2. (**a**) The measured output depending on repeated application of 10 kPa; (**b**) the frequency response in the frequency range of 3~500 Hz, where the inset shows the measurement setup; (**c**) the fast Fourier transform of the sensor output obtained during surface scan of a silicon grating structure with 800 μm period, where the left inset shows the SEM image of grating structure (scale bar is 1 mm) and the right inset shows the real-time measurement data.

Figure 6a shows the output current of SOTS2 during 30 cycles of 10 kPa pressure application. The magnitude of the output had only 3% deviation. Such high stability of output magnitude comes from the reliable contact switching between the top electrode and the spatially digitized resistors [10,29]. The output current levels may be reduced by reducing the device supply voltage for lower power operation. The frequency response was investigated by applying vibrational stimulus using a piezoactuator, with a 3 μm vertical displacement, at the top ridge structure of SOTS, as shown in the inset of Figure 6b. The variation in pressure would result in the time dependent variation in contact area, and therefore the number of resistors making contact. Figure 6b shows the fast Fourier transform data of the SOTS2 output depending on vibrational pressure application at the frequency range of 3~500 Hz. The results show that it was possible to determine the frequency of the applied pressures, and that the response magnitude was 20 dB larger than the noise for the entire frequency range. The fluctuation observed under 5 dB at all frequency regions was assumed to be caused by white noise, the low frequency peak under 10 Hz is from background base line. This demonstrated that at the measured frequency, which was similar to the range of human mechanoreceptor sensing range [31], SOTS showed high sensitivity and the possibility to detect low level surface vibrations.

We also attempted to detect contact sheer signals using SOTS. The PDMS ridge structures on the SOTS surface functions to convert lateral shear into vertical vibrations, similar to human fingerprint structures, The shear force was applied on top of the SOTS2 surface PDMS ridge structures at 1.6 mm/s scanning speed. Figure 6c shows the output signals in the frequency domain and the inset shows the time domain. From the frequency domain, we could identify 2 Hz periodicity, which was a close match to the periodicity predicted by the grating period and scanning speed. Considering the output current level, only one or two resistors were in contact indicating that the applied pressures were in the range of, or slightly above ~0.2 kPa, which demonstrates the high sensitivity of the SOTS at low amplitude vibrational pressures.

## 3. SOTS Array

### 3.1. Concept and Fabrication

**Figure 7:**
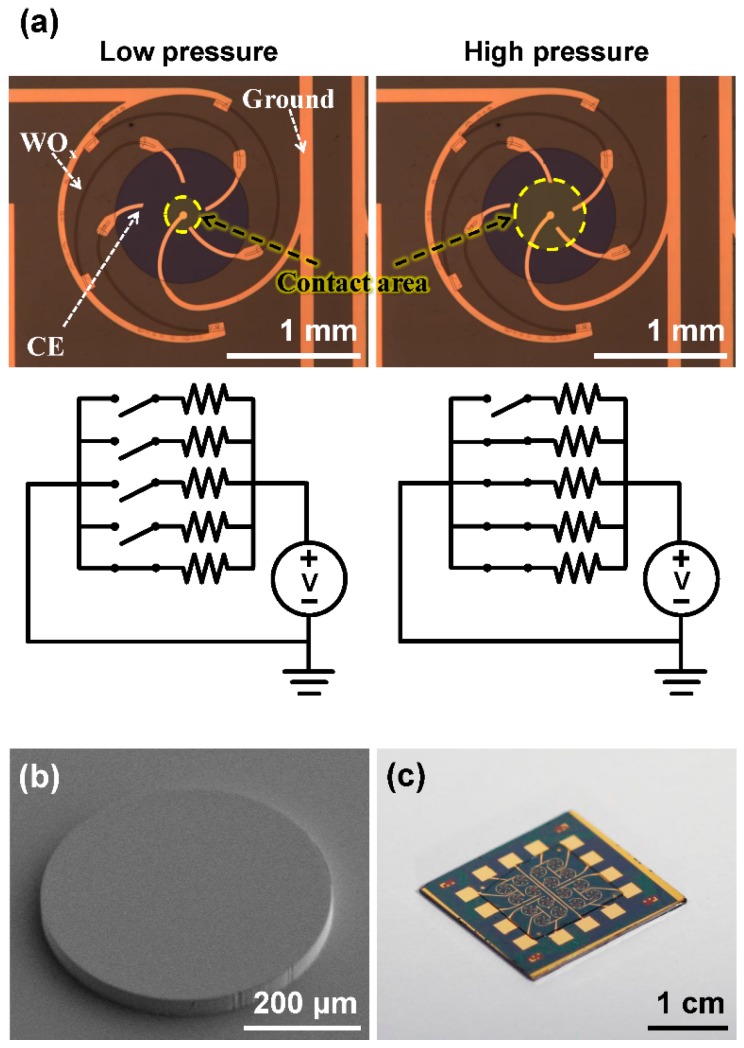
The images and operating mechanism of the 4 × 4 SOTS array. (**a**) The contact area when low and high pressure are applied, and equivalent circuits diagrams; (**b**) the SEM image of PDMS bump structure with 375 μm diameter and 40 μm height; and (**c**) the optical image of the 4 × 4 SOTS array.

To detect surface pressure distribution, an array of sensors are required. Thus, we have improved the basic sensor design to facilitate multiple device integration by reducing device area and removing contacts to the top electrodes. Figure 7a shows the optical images and the operating mechanism of the revised SOTS. First, the Pt top electrodes were not separated as shown in Figure 1 and were used to complete the circuit connection between the ground and spatially separated contact electrodes [27]. This removed the additional step to fabricate contact electrodes on the SU-8 spacer surface (as in Figure 2i), simplifying the device assembly process. Second, the contact electrodes were arranged in a spiral to reduce the overall size of the individual sensor while maintaining high resistance of the resistors to within 4 mm^2^. And finally, the WO_x_ resistors with 7.4 kΩ were placed under the spacer enhancing mechanical and chemical stability. The diameter of the spacer pit was 1 mm, and the gap between the spatially digitized contact electrodes (CEs) were 70 μm, preventing simultaneous contacts at low pressure levels. The diaphragm layer was replaced with a 100 μm thick PDMS instead of PET, to lower the Young’s modulus which gives enhanced sensitivity to low level pressures. Figure 7b shows the SEM image of the bump structure on top of the PDMS layer. The bump improves the pressure sensitivity by localizing the contact stimulus to the pit area [32,33]. Figure 7c shows an optical image of the integrated 4 × 4 SOTS array on a single chip. The overall size of the sensor array was 2 × 2 cm^2^, and 16 unit sensors were located within 1 × 1 cm^2^.

**Figure 8:**
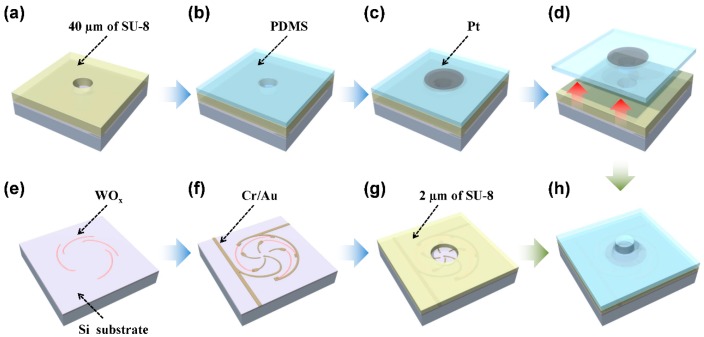
The fabrication process of the 4 × 4 SOTS array. (**a**) SU-8 master for bump structure molding; (**b**) PDMS coating and curing; (**c**) sputter deposition of top Pt electrode; (**d**) peel-off; (**e**) sputter deposition of WO_x_ resistors; (**f**) deposition of spatially digitized electrodes and contact electrodes; (**g**) Su-8 spacer formation; (**h**) bonding of the top layer and the bottom substrate.

Figure 8 shows illustrations of the fabrication process for the 4 × 4 SOTS array. For the top layer we used a molding technique starting with a 40 μm thick SU-8 master (Figure 8a). 100 μm thick PDMS was coated and cured (Figure 8b). After sputtering 100 nm Pt electrode, the top layer with a bump structure was peeled off (Figure 8c,d). To fabricate the bottom substrate, the pattern of resistors was formed by using optical lithography, and tungsten sputtered in oxygen atmosphere (Figure 8e). The spatially digitized electrode and contact electrode were patterned, and 20 nm Cr/ 100 nm Au was evaporated (Figure 8f). Then the 2 μm thick SU-8 spacer was formed using optical lithography (Figure 8g). Finally the top layer and bottom substrate was aligned and bonded (Figure 8h).

### 3.2. Operating Characteristics

**Figure 9:**
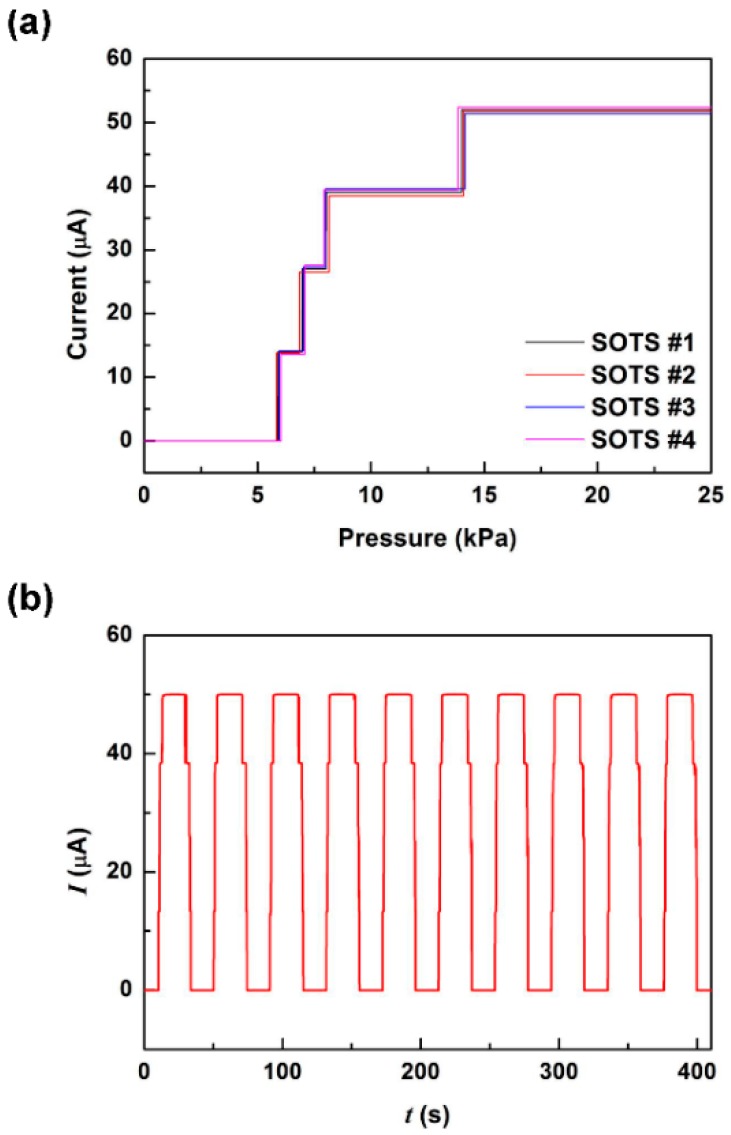

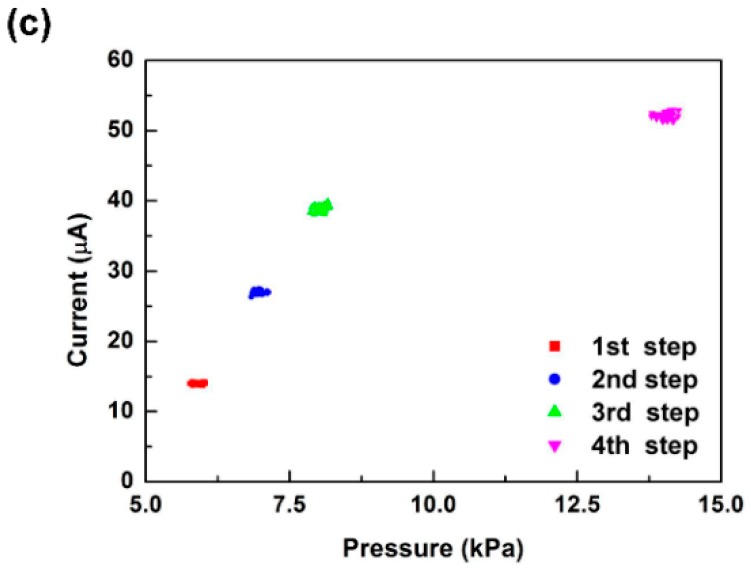
The operating characteristics of 4 × 4 SOTS arrays. (**a**) The pressure dependent measurement results of four unit sensors showing the fully stepped output characteristics and operating uniformity; (**b**) the output depending on repeated application of 20 kPa of contact pressure; (**c**) the distribution of pressure threshold and current magnitude at each current step of 32 sensors in two array devices.

Figure 9a shows the response of four sensors in the array with 0.1 V supply voltage. As the applied pressure gradually increased, we were able to observe four current steps of about 13 μA at designated pressures between 6~14 kPa. The nonlinear sensor response results from the nonlinearly deflecting diaphragm making contact with uniformly spaced resistors. The sensors showed reproducible results under repeated pressure cycles (Figure 9b) with about 0.5% distribution in current. It can be observed that results from 32 sensors from two array devices demonstrates relatively high uniformity of threshold pressure and current magnitude at each pressure level step (Figure 9c).

### 3.3. Read-Out Circuit

**Figure 10:**
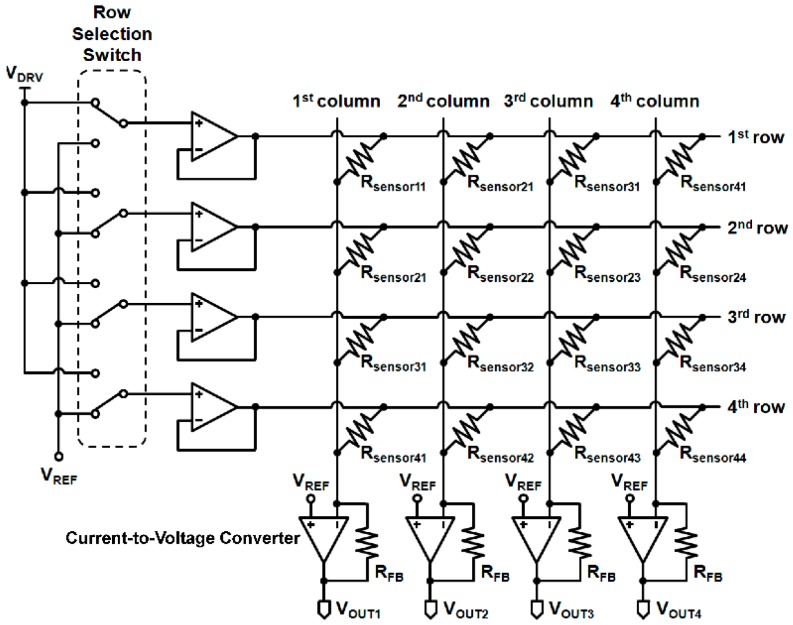
The read-out circuit for the 4 × 4 SOTS array.

A readout circuit was prepared to read all of the 16 sensors without any cross-talk, in sequence (see Figure 10). A single-pole, single–throw (SPDT) switch (ADG733, Analog Devices Inc., Norwood, MA, USA) was used as a row selection switch, and high precision amplifier (LMP7701, Texas Instruments, Dallas, TX, USA) was used as an amplifier. When the driving voltage (*V_DRV_*), the reference voltage (*V_REF_*) and the feedback resistor (*R_FB_*) were at 0 V, 0.5 V and 7.4 kΩ, respectively, the voltage output (*V_OUT_*) was given as:

(5) $$V_{OUT}=V_{REF}-(V_{DRV}-V_{REF})\frac{R_{FB}}{R_{sensor}}=0.5+0.5\frac{7.4k}{R_{sensor}}$$

where, *R_sensor_* is the measured resistance of a unit sensor. The five levels of detectable pressures were converted to the output voltage of 0.5, 1, 1.5, 2, and 2.5 V.

### 3.4. Pressure Mapping Capabilities

**Figure 11:**
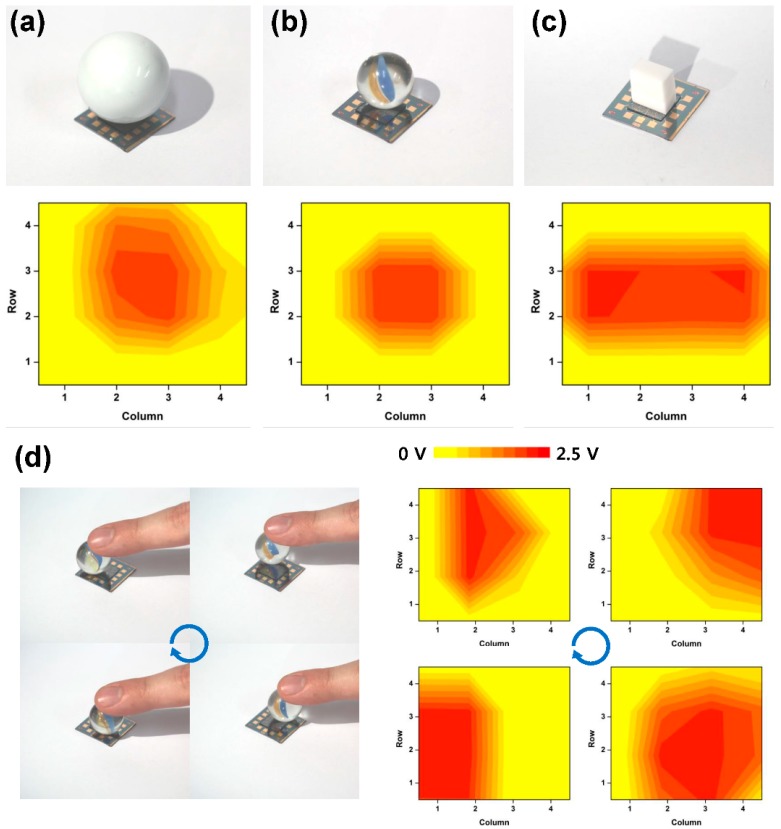
The pressure (voltage) distribution map converted from the SOTS array’s read-out voltage, when spheres with (**a**) 20 mm and (**b**) 8 mm radius; and (**c**) rectangular eraser with 4 × 10 mm contact area were pressed on the 4 × 4 SOTS array; (**d**) The voltage color map of SOTS array when the sphere was in circular motion. All optical images show the SOTS array before wiring.

The surface pressure distribution sensing characteristics of the SOTS array is shown in Figure 9. The optical images were taken before the sensor was wired for measurement. The test objects placed on the sensor array surface were spheres with radius of 8 and 20 mm, and a rectangular eraser with 4 × 10 mm^2^ contact area which weighed 40, 5 and 2 g, respectively. Once the objects were placed on the sensor array surface, the five levels of detectable pressures were converted to the output voltage by the readout circuit and the intermediate levels between the sensor positions were interpolated based on the measured levels at each sensor point. The detected pressurized contact area for the hard spheres in Figure 11a,b became wider due to the PDMS layer deformation resulting in the distribution of vertical pressure laterally. Considering that 2 g placed over 1 cm^2^ area equals 200 Pa, the measured distribution of 8~14 kPa for the assumed contact points shows that the bump structure acted to localize the contact pressure enabling detection of such low contact pressures. This demonstrated that the SOTS array was able to measure the distribution of contact pressures between 0~14 kPa, within the 1 × 1 cm^2^ surface area. Figure 11d shows detection of dynamic pressure distribution using the readout circuit (1 V supply voltage), which demonstrates that the SOTS array was also capable of detecting dynamic changes in contact pressures.

The power consumption levels of SOTS may be tailored by controlling the supply voltage and the value of resistors. If we compare the power consumption levels between SOTS2 and the integrated SOTS, by reducing the supply voltage and increasing resistor value the output power during operation was reduced by 1/1000 to ~5 μW at saturation pressures. It may be possible to further reduce the output power by utilizing the stepped nature of the output current. Since a designated current level determines the measured pressure, the output current may be reduced to levels where the measurement noise does not exceed the step current distinguishing each detected pressure levels. The sensor is in open circuit mode when no pressure was detected and the current fluctuation was at 10^−12^ A levels meaning that the power consumption may be lowered to 10^−12^ W levels while still maintaining high SNR levels. The ability to distinguish small differences in pressures at low contact pressure ranges may allow SOTS to function as a minimum pressure level indicator for systems that utilizes predesignated contact pressures as triggers for a desired action command. Also, high sensitivity to low frequency contact vibrations may also allow SOTS to determine the differences in contact shear characteristics of materials with varying surface roughness enabling robotic tactile interfaces and electronic skin prosthesis.

## 4. Conclusions

We have developed a tactile pressure sensor that produces stepwise output currents when contact pressures are applied. The sensor’s operation relies on the pressure dependent deflection of the polymer diaphragm making contact with resistive current paths on the substrate surface. The stepped output current depended on the number of short-circuited resistors, which reflected the tactile pressure level. The SOTS showed that they can have variable sensitivity and sensing range just by adjusting the number and spacing between the resistive current paths. They also showed SNR above 20 dB at contact pressures below 15 kPa. A 4 × 4 SOTS array with a surface bump structure was fabricated to demonstrate 1 × 1 cm^2^ areal pressure mapping capabilities.
